# The Year-Book of Treatment

**Published:** 1891-06

**Authors:** 


*The Year-Book of Treatment.* London: CASSELL & CO. 1891.

*The Medical Annual.* Bristol: WRIGHT & CO. 1891.

We have again to welcome the appearance of these useful Annuals. Both are improvements on previous numbers; *The Medical Annual* more evidently so than *The Year-Book.*

The value of *The Year-Book* is still diminished by the fact that the reports of papers are not always neutral, but are occasionally disfigured by personal opinions and criticisms of the abstractor. Now we—the public—care nothing for the opinions of the abstractor: we want the opinions of the men who wrote the papers—men whose repute is usually superior to that of their critics, and who have no opportunity of replying to these criticisms. Of one thing the writers of these abstracted papers might properly complain; and that is, when their papers are not fairly presented. Three such we have happened to note. And we like to know that an abstract is done from an original paper. An abstract of an abstract is not likely to be trustworthy; and our belief in it will not be enhanced if it is recorded as being directly from the original, when we know it could not have been so. For instance, an abstract whose only acknowledged source is from a journal written in a language a knowledge of which the abstractor does not add to his many accomplishments, is not likely to command our respectful attention. Has *The Year-Book* at its head a man of acknowledged editorial capacity and firmness?

*The Medical Annual* has none of these faults, and shines by the possession of antithetic virtues. Besides the usual summaries and references, arranged alphabetically, there are several excellent articles on special subjects of present interest. Conspicuous among these last may be mentioned Dr. Long Fox's exhaustive article on "The Hand as a Diagnostic Factor in Nerve Disease," and Dr. Wethered's article on "The Sputum as an Aid to Diagnosis."

